# Implications of a new clinical classification of acute myocardial infarction

**DOI:** 10.1093/ehjacc/zuaf065

**Published:** 2025-04-30

**Authors:** Alexander J F Thurston, Jasper Boeddinghaus, Andrew R Chapman, Bertil Lindahl, Nicholas L Mills

**Affiliations:** BHF/University Centre for Cardiovascular Science, University of Edinburgh, Edinburgh, UK; Cardiovascular Research Institute Basel (CRIB) and Department of Cardiology, University Hospital Basel, University of Basel, Basel, Switzerland; BHF/University Centre for Cardiovascular Science, University of Edinburgh, Edinburgh, UK; Department of Medical Sciences, Uppsala University, Uppsala, Sweden; BHF/University Centre for Cardiovascular Science, University of Edinburgh, Edinburgh, UK

We thank the correspondents for their interest in our original article.

We proposed a new clinical classification of myocardial infarction to ensure the diagnosis is based on objective criteria and has clear implications for patients.^1^ This is particularly important in those presenting with supply-demand imbalance secondary to an alternative acute condition where the adoption of the diagnosis of type 2 myocardial infarction has been inconsistent.^2^ In this setting, the diagnosis of secondary myocardial infarction would require identification of a substrate (obstructive coronary disease) or a consequence of ischaemia (new ventricular impairment or scar).

In our study, patients underwent systematic imaging, and those with atherothrombosis were adjudicated as spontaneous myocardial infarction.^3,4^ Patients in whom the diagnosis of spontaneous or secondary myocardial infarction were excluded did not have a benign course, with half of the patients having imaging evidence of alternative cardiac diagnoses that increase cardiovascular risk. Given our systematic imaging protocol, unrecognized atherothrombosis is unlikely.

**Figure 1:**
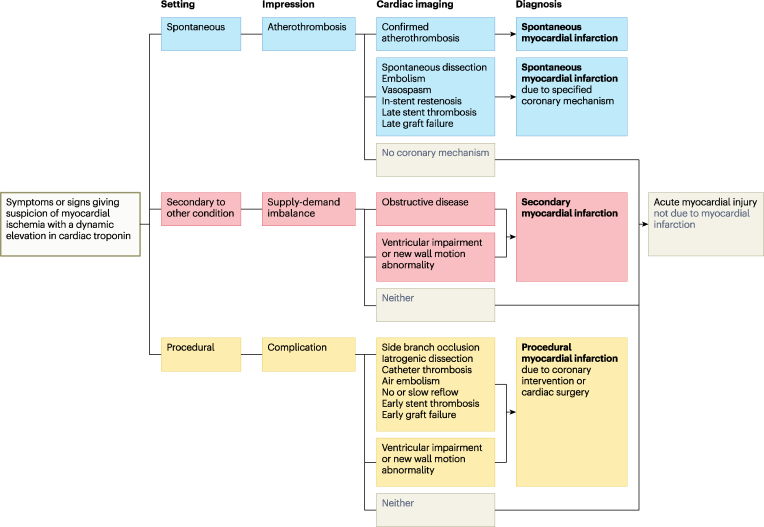
Proposal for a clinical classification of myocardial infarction. Reproduced from Lindahl & Mills, *A new clinical classification of acute myocardial infarction* Nature Medicine 2023.^1^

We respectfully disagree that the new classification is agnostic to the coronary aetiology. In patients with a spontaneous presentation, the classification encourages identification of the underlying acute coronary mechanism and incorporation into the diagnosis (e.g. ‘spontaneous myocardial infarction due to SCAD’) (*Figure 1*). This would facilitate communication with patients, tailored treatment and research into understudied non-atherothrombotic mechanisms of myocardial infarction.

We concur timely adjunctive investigations are invaluable. A strength of the new classification is the emphasis on imaging to define the underlying coronary, cardiac or systemic pathology, guide treatment and improve outcomes for patients.
